# Cerebrospinal fluid neurofilament light chain predicts short-term prognosis in pediatric Guillain-Barré syndrome

**DOI:** 10.3389/fneur.2022.972367

**Published:** 2022-08-22

**Authors:** Mei Jin, Kang Liu, Libo Zhao, Jing Liu, Ziwei Zhao, Yifan Zhao, Suzhen Sun

**Affiliations:** Department of Pediatric Neurology, Children's Hospital of Hebei Province, Shijiazhuang, China

**Keywords:** Guillain-Barré syndrome, children, neurofilament light chain, Hughes functional grading, prognosis

## Abstract

**Introduction:**

To study cerebrospinal fluid neurofilament light chain (CSF-NfL) levels as a prognostic biomarker in pediatric Guillain-Barré syndrome (GBS).

**Methods:**

Prospective study enrolling 26 pediatric GBS patients and 48 healthy controls (HCs) from neurology units between 2017 to 2021. The CSF-NfL levels were measured by enzyme-linked immunosorbent assay. The children's disability levels were evaluated using Hughes Functional Score (HFS) at nadir, 1 month, and 6 months after onset. The receiver operating characteristic (ROC) curve derived from logistic regression (with age as a covariate) was used to assess the prognostic value of CSF-NfL on the possibility of walking aided at 1 month after symptom onset.

**Results:**

The mean CSF-NfL levels were significantly increased in GBS patients (111.76 pg/mL) as compared to that in HCs (76.82 pg/mL) (*t* = 6.754, *p* < 0.001). At follow- up, the mean CSF-NfL levels after treatment (65.69 pg/mL) declined significantly (*t* = 6.693, *p* < 0.001). CSF-NfL levels upon admission were significantly associated with the HFS at nadir (*r*_*s*_ = 0.461, *p* = 0.018). Moreover, the mean CSF-NfL levels in GBS patients with poor prognosis (130.47pg/mL) were significantly higher than that in patients with good prognosis (104.87pg/mL) (*t* = 2.399, *p* = 0.025). ROC curve analysis of the predictive value of CSF-NfL levels with respect to the inability to walk unaided within 1 month showed a significant difference (area under the curve: 0.857,95% confidence interval 0.702-1.000; *p* = 0.006).

**Conclusion:**

CSF-NfL levels were increased in pediatric GBS patients. High CSF-NfL level predicted worse motor function, and was strongly associated with poor short-term prognosis of pediatric GBS. We propose a biomarker for early prediction of outcome in pediatric GBS, which would be applicable for clinical practice and efficacy of treatment in the future.

## Introduction

Guillain–Barré syndrome (GBS) is currently the most common cause of acute flaccid paralysis in children and is thought to be provoked by an aberrant immune response. Potential triggering factors include viruses such as severe acute respiratory syndrome coronavirus-2 (SARS-CoV-2), Zika virus, cytomegalovirus (CMV), Epstein–Barr virus (EBV), and influenza virus, bacteria such as *Campylobacter Jejuni, Mycoplasma Pneumoniae* and other factors such as vaccinations, surgery, and malignancy (1). Since GBS associated with COVID-19 is being increasingly reported, and given the current impact of the COVID-19 pandemic, it is important to conduct SARS-CoV-2 reverse transcription polymerase chain reaction (RT-PCR) testing with naso- or oropharyngeal swab in all patients with suspected GBS to rapidly isolate cases. The diagnosis of GBS is mainly based on clinical history and presentation, and ancillary investigations such as cerebrospinal fluid (CSF) examination and electrophysiological studies (2). However, making a diagnosis of pediatric Guillain-Barré syndrome can be challenging, and it is highly dependent on ancillary examinations. Electrophysiological studies play an important role in early diagnosis and determining the subtypes of GBS (3). However, the electrophysiological measurements can be normal in the early course of the disease, and hence they might not meet the electrophysiological diagnostic criteria of GBS. Therefore, there is an urgent need for a sensitive and specific biomarker that could identify GBS at the acute stage.

Many studies have shown that patients with GBS differ considerably in their clinical manifestation and prognosis (4, 5). Children with GBS tend to have a good prognosis (6). However, a minority of patients were unable to walk independently 6 months after onset. Therefore, it is critical to identify poor prognoses early on for pediatric GBS patients for implementing effective treatment strategies to avoid irreversible nerve degeneration. Some prognostic methods, such as the modified Erasmus GBS outcome score and Erasmus GBS respiratory insufficiency score can be applied to adult patients with GBS (7, 8), however, whether they are suitable for children with GBS remains unknown. Therefore, it is urgently necessary to discover an objective and valid biomarker that could predict the outcome of GBS in children.

As the most important axonal damage biomarker, neurofilament levels have emerged as important in neurological disorders, such as multiple sclerosis (9), Alzheimer's disease (10), and Guillain–Barré syndrome. Some studies have shown that the CSF and serum concentrations of neurofilament heavy chain levels (NfH) were increased in children with GBS (11), and other studies have demonstrated that high serum neurofilament light chain levels (sNfL) were also increased and associated with poor prognosis in adult GBS (12), however, the correlation of CSF-NfL levels and outcome in children with GBS were not analyzed in these studies. Thus, in the present study, we aimed to: (1) determine the CSF-NfL levels by enzyme-linked immunosorbent assay (ELISA) in healthy children and pediatric GBS patients; (2) analyze the value of CSF-NfL levels in early diagnosis and therapeutic efficacy in pediatric GBS; and (3) examine the association between CSF-NfL levels and clinical features as well as the outcome of Guillain-Barré syndrome in children.

## Materials and methods

### Subjects

We enrolled 26 pediatric GBS patients (under 14 years of age) who were admitted to our hospital between September 2017 and January 2021. Patients fulfilling levels 1 or 2 of the Brighton criteria of GBS (13) were included in the study. Patients with Miller Fisher syndrome and other causes of neuropathies, such as spinal anterior horn lesion caused by infection of poliovirus or enterovirus 71, acute transverse myelitis, chronic inflammatory demyelinating polyneuropathy, were excluded. The control group consisted of 48 children who visited our hospital because of psychiatric disorders, migraine, and benign intracranial hypertension. They did not present with any known axonal damage or the presence of objective clinical signs after extensive diagnostic evaluation. All the participants provided written informed consent for the use of their CSF for research purposes. This study was approved by the Ethics Committee of the Children's Hospital of Hebei Province.

### Methods

#### CSF samples and analytical methods

CSF samples of GBS patients were collected upon admission and 2 weeks after the treatment. All samples were collected into a polypropylene tube and stored at −80°C until the analysis. Samples were analyzed by two investigators blinded to clinical data using the Nf-light kit (ELISA kits: Uman Diagnostics NF-light^®^). Sample processing was carried out according to the manufacturer's instructions and protocol. All samples were measured in duplicates. The mean intra- and inter-assay coefficients of variation for mean CSF-NfL level (111.76 pg/mL) were 1.9and 4.5%, respectively.

#### Clinical data and ancillary investigations

(1) Limb disability was assessed by Hughes Functional Scale (HFS) at nadir, 1 month, and 6 months after onset. Patients with HFS ≥ 3 were categorized as poor outcomes, and patients with HFS <3 were categorized as good outcomes. Other clinical manifestations, such as facial paralysis, paraesthesias, autonomic dysfunction, and mechanical ventilation were also analyzed. (2) The pleocytosis and protein levels of CSF were measured within 4 weeks of weakness onset. (3) Electrophysiologic examinations were performed at least twice and patients were classified into acute inflammatory demyelinating polyneuropathy (AIDP) and acute motor axonal neuropathy (AMAN) according to the Hughes electrodiagnostic criteria (14).

#### Statistical analyses

Statistical analyses were performed using the IBM SPSS Statistics 24. Categorical data were shown as proportions, and continuous data were shown as mean ± SD or medians with IQR. Differences in proportions were tested by χ^2^ tests. The continuous variables were tested by the *t*-test or Mann–Whitney U test. The associations between basal characteristics and CSF-NfL levels were analyzed using Pearson correlation or Spearman's correlation coefficient. The receiver operating characteristic (ROC) curve derived from logistic regression (with age as a covariate) was used to evaluate the prognostic value of CSF-NfL on the probability of walking dependently. Statistical significance was set at 0.05.

## Results

### CSF-NfL in pediatric GBS patients and healthy controls

**Figure 1:**
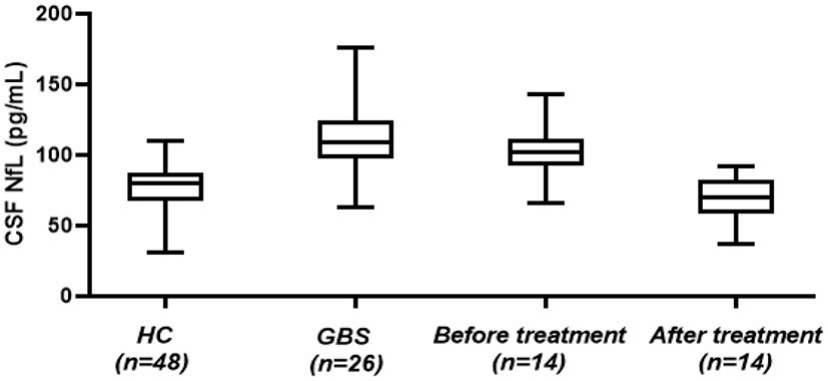
CSF-NfL levels in GBS patients and healthy controls. Box plots indicate median and IQR with whiskers extending 1.5 times the IQR. CSF-NfL, cerebrospinal fluid neurofilament light chain; GBS, Guillain–Barré syndrome; HC, healthy control.

A total of 26 children patients (mean age 6 years; 14 males) were recruited. The mean CSF-NfL level for the GBS patients upon admission was 111.76 ± 26.33 pg/mL. Forty-eight healthy age-matched controls (mean age 7 years; 30 males) were included, and their mean CSF-NfL level was 76.82 ± 17.96 pg/mL. CSF-NfL levels were significantly higher in the GBS patients than in healthy controls (*t* = 6.754, *p* < 0.001) (Table 1; Figure 1).

**Table 1:** CSF-NfL in patients with GBS upon admission and healthy controls.

**Variables**	**GBS patients (*n =* 26)**	**Healthy controls (*n =* 48)**	**Statistic values**	***P-value***
Age, years, mean±SD	6 ± 3.4	7 ± 2.6	*t =* 1.561	0.123
Male, *n* (%)	14(53.8)	30(62.5)	χ^2^ = 0.524	0.469
NfL,pg/mL, mean±SD	111.76 ± 26.33	76.82 ± 17.96	*t =* 6.754	<0.001

At follow-up, 14 patients were recruited, and the mean CSF-NfL levels before and after treatment were 101.85 ± 19.12 pg/mL and 65.69 ± 16.71 pg/mL, respectively. The CSF-NfL levels after treatment declined significantly (*t* = 6.693, *p* < 0.001) (Figure 1). The CSF-NfL levels were not significantly different between GBS patients after treatment and the healthy controls (*t* = 2.069, *p* = 0.053) (Figure 1).

### Relationship between CSF-NfL levels and baseline clinical features in pediatric GBS

**Figure 2:**
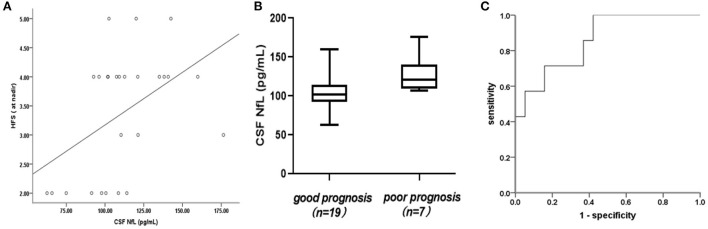
**(A)** Association of cerebrospinal fluid neurofilament light chain (CSF-NfL) concentrations upon admission with the Hughes Functional Score (HFS) calculated at nadir with a Spearman's correlation coefficient *r*_*s*_ of 0.461 (*p* = 0.018). Each dot in the scatter plot represents a sample. **(B)** CSF-NfL levels on admission in patients who were good or poor prognoses at 1 month after symptom onset. Box plots indicate median and IQR with whiskers extending 1.5 times the IQR. **(C)** Receiver operating characteristic (ROC) curve derived from logistic regression (with age as a covariate) was used to analyze the potential predictive value of CSF-NfLlevels with respect to unable to walk unaided (Hughes grade of 3 and more).

CSF-NfL levels upon admission were significantly associated with the Hughes Functional Score (HFS) calculated at nadir (*r*_*s*_ = 0.461, *p* = 0.018) (Figure 2A). Meanwhile, CSF-NfL levels were correlated with CSF protein levels (*r*_*s*_ =0.392, *p* = 0.048). Other clinical features and CSF-NfL levels are summarized in Table 2.

**Table 2:** Relationship between CSF-NfL levels and basal characteristics in pediatric GBS.

**Basal characteristics (*n =* 26)**	**CSF-NfL levels pg/mL**	**Statistic values**	***P-value***
Age, years, mean±SD	6 ± 3.4	*r =* 0.119	0.561
Gender, *n* (%)		*r =* 0.289	0.152
Male	14(53.8)		
Female	12(46.2)		
Preceding event, *n* (%)		—	0.256
Respiratory infection	16(61.5)		
Diarrhea	1(3.8)		
None	9(34.6)		
From onset to nadir, days, median (IQR)	7.5(3.75–12)	*r_*s*_* = 0.249	0.220
Hughes score at nadir, grade, median (IQR)	4(2–4)	*r_*s*_* = 0.461	0.018
**Neurological symptoms**, ***n*** **(%)**			
Facial paralysis	5(19.2)	*r =* 0.023	0.910
Bulbar paralysis	6(23.1)	*r =* 0.107	0.603
Neuropathic pain	17(65.4)	*r =* 0.080	0.697
Autonomic dysfunction, *n* (%)	7(26.9)	*r =* 0.215	0.292
Mechanical ventilation, *n* (%)	14(53.8)	*r =* 0.023	0.911
EMG variants, *n* (%)		*r =* 0.187	0.359
AIDP	23(88.5)		
AMAN	3(11.5)		
Proteins in CSF, g/L, median (IQR)	0.93(0.68–1.31)	*r_*s*_* = 0.392	0.048
Pleocytosis, 10^6^/L, median (IQR)	4(2–9.25)	*r_*s*_* = 0.124	0.546
Treatment, *n* (%)		*r =* 0.087	0.671
IVIg	23(88.5)		
IVIg + PLEX	3(11.5)		
Hughes score at 1 month after onset, grade, median (IQR)	1(1–3)	*r_*s*_* = 0.433	0.027
Hughes score at 6 month after onset, grade, median (IQR)	0	*r_*s*_* = 0.107	0.602
Duration of hospitalization, days, mean ± SD	25.08 ± 11.2	*r =* 0.243	0.232
Prognosis, *n* (%)		*r =* 0.440	0.025
Good	19(73.1)		
Poor	7(26.9)		

GBS, Guillain-Barré syndrome; AIDP, acute inflammatory demyelinating polyneuropathy; AMAN, acute motor axonal neuropathy; IVIg, Intravenous immunoglobulin; PLEX, plasma exchange; r, Pearson correlation; r_s_, Spearman's correlation coefficient.

There was no association between CSF protein levels and prognosis in GBS patients (*r*_*s*_ = 0.002, *p* = 0.991), and receiver operating characteristic (ROC) curve analysis of the predictive value of CSF protein levels showed no statistical difference (area under the curve [AUC]: 0.594, 95% confidence interval [CI] 0.363–0.825; *p* = 0.470).

In this study, the mean CSF-NfL levels in GBS patients with good or poor prognosis were 104.87 ± 24.18 and 130.47 ± 24 pg/mL, respectively, furthermore, this difference was significant (*t* = 2.399, *p* = 0.025) (Figure 2B). A correlation between CSF-NfL and age in adults has been reported previously. We performed an analysis of covariance, with age as a confounder. The mean age in the GBS group was 6 ± 3 years and 7 ± 3 years in the control group. Age and CSF-NfL levels were significantly associated in the control group (*r* = 0.0.841, *p* < 0. 001), which was not the case for the GBS patients (*r* = 0.119, *p* = 0.561). Analysis of covariance suggested no influence of age on CSF-NfL concentrations (*F* = 2.522, *p* = 0.126), whereas the age-adjusted group difference was preserved (*p* = 0.009). ROC curves derived from logistic regression (with age as a covariate) were used to analyze the potential predictive value of CSF-NfL levels with respect to unable to walk unaided (Hughes grade of 3 and more) within 1 month, which showed a statistically significant difference (area under the curve [AUC]: 0.857,95% confidence interval [CI] 0.702–1.000; *p* = 0.006) (Figure 2C).

## Discussion

Guillain-Barré syndrome (GBS) is one of the most common causes of acute flaccid paralysis in children, and is characterized by progressive, relatively symmetrical limb weakness with or without paresthesia (15). The prognosis of GBS tends to be good after effective treatments, however, about one-fifth of the patients are unable to walk independently at 6 months after symptom onset, and about 7% of patients die (16). Many studies have shown that pediatric GBS has a good prognosis (17, 18). In the present study, seven (7/26, 26.9%) and 19 patients (19/26, 73.1%) showed poor and good prognoses, respectively, at 1 month after symptom onset, whereas none of the patients was unable to walk independently at 6 months after symptom onset. Therefore, a convenient and reliable biomarker to predict the clinical prognosis of pediatric GBS is essential for patients.

Neurofilament light protein (NfL) is one of the most important intracellular neuronal skeletal proteins and is secreted into CSF during axonal damage (19). In the literature to date, the potential value of NfL as a biomarker for axonal damage has been discussed in multiple neurological disorders, such as amyotrophic lateral sclerosis (20), chronic inflammatory demyelinating polyneuropathy (21), cerebral vasculitis (22). Patients with these conditions have elevated NfL levels as compared to healthy individuals. This is consistent with the results of our study, wherein mean CSF-NfL levels of pediatric GBS patients were significantly higher than those of healthy controls. Moreover, at follow-up, mean CSF-NfL levels after treatment significantly declined along with the amelioration of the disease. These findings indicate that CSF-NfL levels might become an objective biomarker for early diagnosis and therapeutic efficacy.

CSF albuminocytological dissociation phenomenon could further assist the diagnosis of GBS (23). CSF protein elevation reflected the damage of the blood-nerve and blood-brain barrier and the subsequently increased permeability. GBS patients with higher CSF-NfL levels had more severe demyelination and axonal damage than GBS patients with lower CSF-NfL values, which also manifested as a more severe motor disability. In the present study, CSF-NfL levels were significantly correlated with protein levels and the Hughes Functional Score (HFS) at nadir. All these findings signify that higher CSF-NfL levels could be used to predict worse motor function and more clinical severity.

Higher CSF-NfL levels in adult GBS had also been associated with a poorer clinical prognosis (24). In the present study, CSF-NfL levels in GBS patients with poor prognosis were significantly higher than that in patients with good prognosis. Further, ROC curve analysis demonstrated the potential predictive value of the CSF-NfL levels. This showed that higher CSF-NfL levels were strongly correlated with poor short-term prognosis of GBS, and served as an independent factor associated with the inability to walk. Therefore, higher CSF-NfL levels could also alert physicians in the early stage of the disease to ensure active and effective treatments to improve the prognosis and shorten the course of the disease.

Our study was also subject to some limitations. First, the association of CSF-NfL levels and electrophysiological subtypes was not evaluated because of a smaller number of cases. Secondly, this was a small prospective study that recruited 26 pediatric GBS patients. We will further conduct some prospective clinical studies on the prognosis of children with GBS based on the present study.

In conclusion, CSF-NfL levels are increased in pediatric GBS patients, higher CSF-NfL predicted worse motor function and were strongly associated with poor short-term prognosis of pediatric GBS. We proposed a biomarker for early prediction of outcome in the pediatric GBS, applicable for clinical practice and future treatment efficacy.

## Data availability statement

The original contributions presented in the study are included in the article/supplementary material, further inquiries can be directed to the corresponding author/s.

## Ethics statement

This study was approved by the Ethics Committee of the Children's Hospital of Hebei Province. Written informed consent to participate in this study was provided by the participants' legal guardian/next of kin.

## Author contributions

LZ and YZ collected serum and CSF samples. JL acquired the electrophysiological data. ZZ completed the statistical analysis. MJ designed the experiments, interpreted the results, and drafted the initial manuscript. KL reviewed the data and revised the manuscript. SS revised the initial draft and wrote the final manuscript. All authors contributed to the article and approved the submitted version.

## Funding

This work was supported by grants from the Medical Science Research Key Project Plan of Hebei Province in 2020(20200223).

## Conflict of interest

The authors declare that the research was conducted in the absence of any commercial or financial relationships that could be construed as a potential conflict of interest.

## Publisher's note

All claims expressed in this article are solely those of the authors and do not necessarily represent those of their affiliated organizations, or those of the publisher, the editors and the reviewers. Any product that may be evaluated in this article, or claim that may be made by its manufacturer, is not guaranteed or endorsed by the publisher.
